# Development and characterization of highly polymorphic long TC repeat microsatellite markers for genetic analysis of peanut

**DOI:** 10.1186/1756-0500-5-86

**Published:** 2012-02-03

**Authors:** Selma E Macedo, Márcio C Moretzsohn, Soraya C M Leal-Bertioli, Dione MT Alves, Ediene G Gouvea, Vânia CR Azevedo, David J Bertioli

**Affiliations:** 1 Institute of Biological Sciences, Campus Darcy Ribeiro, University of Brasilia, CEP 70.910-900 Brasília, DF, Brazil; 2 Embrapa Genetic Resources and Biotechnology, PqEB W5 Norte Final, CP 02372, CEP 70.770-900 Brasília, DF, Brazil; 3 Biotechnology and Genomic Sciences, Catholic University of Brasilia, SGAN 916 Avenida W5, CEP 70.790-160 Brasilia, DF, Brazil

## Abstract

**Background:**

Peanut (*Arachis hypogaea *L.) is a crop of economic and social importance, mainly in tropical areas, and developing countries. Its molecular breeding has been hindered by a shortage of polymorphic genetic markers due to a very narrow genetic base. Microsatellites (SSRs) are markers of choice in peanut because they are co-dominant, highly transferrable between species and easily applicable in the allotetraploid genome. In spite of substantial effort over the last few years by a number of research groups, the number of SSRs that are polymorphic for *A. hypogaea *is still limiting for routine application, creating the demand for the discovery of more markers polymorphic within cultivated germplasm.

**Findings:**

A plasmid genomic library enriched for TC/AG repeats was constructed and 1401 clones sequenced. From the sequences obtained 146 primer pairs flanking mostly TC microsatellites were developed. The average number of repeat motifs amplified was 23. These 146 markers were characterized on 22 genotypes of cultivated peanut. In total 78 of the markers were polymorphic within cultivated germplasm. Most of those 78 markers were highly informative with an average of 5.4 alleles per locus being amplified. Average gene diversity index (GD) was 0.6, and 66 markers showed a GD of more than 0.5. Genetic relationship analysis was performed and corroborated the current taxonomical classification of *A. hypogaea *subspecies and varieties.

**Conclusions:**

The microsatellite markers described here are a useful resource for genetics and genomics in *Arachis*. In particular, the 66 markers that are highly polymorphic in cultivated peanut are a significant step towards routine genetic mapping and marker-assisted selection for the crop.

## Background

Peanut (*Arachis hypogaea *L.) is an oil crop of great importance in the tropics: in Africa, its production is comparable to all other grain legumes put together, and in Asia it provides about the same number of calories as soya (FAO, 2009). It has a narrow genetic base due to its recent origin event of tetraploidization [1,2], and this has hindered the application of molecular breeding in this crop.

Microsatellites or simple sequence repeats (SSRs) are useful molecular markers, are abundant, highly dispersed through the genomes of eukaryotes, and locus specific. In addition they are the ideal markers for genotyping allotetraploid species, such as peanut, since they are usually co-dominant and multi-allelic. They are considered suitable as tools for genetic diversity studies, genetic linkage mapping, and for use in plant breeding programs [3].

Over the past years several research groups have put considerable effort into developing SSR markers for the genus *Arachis *in general and cultivated peanut in particular. Now about 5,000 SSR markers have been published [4-21]. These markers have been mainly used for diversity studies of germplasm, and for genetic mapping ([10,11,22-28]. However, in spite of the number of markers available, the very low polymorphism observed within cultivated germplasm requires large-scale marker screening for the identification of sufficient polymorphic markers even for low density genetic maps in populations derived from cultivated × cultivated crosses. For example, in spite of extensive marker screening, the published SSR-based maps of cultivated peanut have only 131, 135 and 175 SSR markers [22,23,28]. In a previous study, we observed that AG/TC microsatellites were more polymorphic than AC/TG ones and that for cultivated germplasm, the highest polymorphism was observed for microsatellites with 21-25 motif repetitions [10]. In this context, we isolated and characterized long repeat AG/TC SSRs as an effort to develop markers with high polymorphism levels for cultivated peanut [10].

## Findings

### Sequencing

Sequences were obtained for 1401 cloned genomic fragments. Most fragments were sequenced in both forward and reverse orientations. Of these 1401 cloned fragments, 65 harbored sequences very similar to already published markers and so were excluded from further analysis (≥ 50% of sequence with BLAST detected similarity with E-value ≤ E-40). Of the remaining sequences, 193 harbored microsatellite repeats. As expected, most were TC/AG repeats. The 143 unique SSR sequences were deposited in GenBank (accession numbers JN887491 to JN887636).

### Design of flanking primer pairs

Of the 193 selected sequences, 135 were appropriate for primer design. Some sequences contained multiple microsatellite repeats that could not be flanked by a single primer pair. Therefore, in total 146 primer pairs were designed. The microsatellites amplified were generally long, the average number of motif repeats being 23.

### Polymorphism levels

All 146 primer pairs amplified PCR products of the expected size. Of these, 85 were polymorphic within the tetraploid samples (including cultivated peanut, a synthetic allotetraploid and an accession of the tetraploid wild species, *A. monticola *(Table 1), and 78 were polymorphic within cultivated germplasm (Table 2).

**Table 1:** *Arachis *genotypes included in this study.

Genotype	Species/Subspecies/Variety	Origin
cv. BR 1	*A. hypogaea *subsp. *fastigiata *var. *fastigiata*	Embrapa Cotton, Brazil

cv. IAC-Caiapó	*A. hypogaea *subsp. *hypogaea *var. *hypogaea*	IAC, Sao Paulo, Brazil

cv. IAC Runner 886	*A. hypogaea *subsp. *hypogaea *var. *hypogaea*	IAC, Sao Paulo, Brazil

cv. IAC-Tatu	*A. hypogaea *subsp. *fastigiata *var. *fastigiata*	IAC, Sao Paulo, Brazil

cv. IAC-5024	*A. hypogaea *subsp. *hypogaea *var. *hypogaea*	IAC, Sao Paulo, Brazil

Mf210	*A. hypogaea *subsp. *fastigiata *var. *fastigiata*	Misiones, Argentina

Mf2352	*A. hypogaea *subsp. *fastigiata *var. *aequatoriana*	Pichincha, Ecuador

Mf2517	*A. hypogaea *subsp. *fastigiata *var. *peruviana*	Guayas, Ecuador

Mf2534	*A. hypogaea *subsp. *hypogaea *var. *hirsuta*	Pichincha, Ecuador

Mf2681	*A. hypogaea *subsp. *fastigiata *var. *vulgaris*	Georgia, USA

Mf3207	*A. hypogaea *subsp. *fastigiata *var. *vulgaris*	Asunción, Paraguay

Mf1625	*A. hypogaea *subsp. *fastigiata *var. *vulgaris*	Minas Gerais, Brazil

Mf3618	*A. hypogaea *subsp. *hypogaea *var. *hirsuta*	La Libertad, Peru

Mf2430	*A. hypogaea *subsp. *fastigiata *var. *peruviana*	Loja, Ecuador

Mf3764	*A. hypogaea *subsp. *fastigiata *var. *peruviana*	Loreto, Peru

Mf3892	*A. hypogaea *subsp. *fastigiata *var. *aequatoriana*	Lima, Peru

Mf3884	*A. hypogaea *subsp. *fastigiata *var. *aequatoriana*	San Martín, Peru

Mf3911	*A. hypogaea *subsp. *hypogaea *var. *hirsuta*	La Libertad, Peru

Mf574	*A. hypogaea *subsp. *hypogaea *var. *hypogaea*	Santa Cruz, Bolivia

Mf788	*A. hypogaea *subsp. *hypogaea *var. *hypogaea*	Tarija, Bolivia

Of106	*A. hypogaea *subsp. *hypogaea *type Xingu	Xingu Indigenous Park, Brazil

Of107	*A. hypogaea *subsp. *hypogaea *type Xingu	Xingu Indigenous Park, Brazil

V14165	*A. monticola*	Jujuy, Argentina

(K30076xV14167)^4x^	*A. ipaënsis *x *A. duranensis*	Embrapa Cenargen, Brazil

Genotypes of *Arachis hypogaea, A. monticola*, and a synthetic allotetraploid used for SSR marker validation and for genetic relationship analysis. *Arachis hypogaea *genotypes represent both subspecies (*hypogaea *and *fastigiata*) and the six botanical varieties (*hypogaea, hirsuta, fastigiata, vulgaris, aequatoriana*, and *peruviana*). (K30076xV14167)^4× ^is a synthetic allotetraploid. IAC is Campinas Agronomic Institute

**Table 2:** List of the 78 polymorphic markers.

Marker name	Major Allele Frequency	Allele number	Gene Diversity
TC13C03	0.4737	3	0.5873

TC13E05	0.4773	8	0.7118

TC14B08	0.5000	5	0.6621

TC14H09	0.9565	2	0.0832

TC15F12	0.3182	12	0.8388

TC16A10a	0.6786	6	0.5102

TC16A10b	0.3333	5	0.7222

TC19A02a	0.6667	2	0.4444

TC19A02b	0.5238	4	0.6270

TC19B07	0.2500	8	0.8472

TC19E01	0.6667	3	0.5000

TC20B05	0.4762	7	0.7086

TC20D05	0.4545	7	0.6374

TC20E08	0.4231	5	0.7278

TC21A09	0.5435	4	0.6011

TC21C03	0.5000	6	0.6791

TC21D06a	0.9286	2	0.1327

TC21D06b	0.3913	3	0.6578

TC21G01	0.2368	6	0.8075

TC22B07	0.9583	2	0.0799

TC22D09	0.8125	3	0.3116

TC22G05	0.4750	5	0.5700

TC22H12	0.5217	6	0.6522

TC23B10	0.4565	5	0.6408

TC23C08a	0.8158	3	0.3144

TC23C08b	0.9348	3	0.1238

TC23D04	0.3043	6	0.7741

TC23E04a	0.3750	6	0.7188

TC23E04b	0.5000	4	0.5564

TC23F04	0.8696	4	0.2382

TC23F09	0.5000	3	0.5450

TC23H10	0.2273	10	0.8636

TC24A06	0.3214	7	0.7985

TC24B05	0.2000	12	0.8850

TC24C06a	0.3182	10	0.8068

TC24D06b	0.6042	5	0.5460

TC24D12	0.5500	3	0.5950

TC24E01	0.9583	2	0.0799

TC24G10	0.5000	5	0.6796

TC25B04	0.4000	7	0.7025

TC25F03	0.5833	5	0.5747

TC25G11	0.3478	7	0.7561

TC27H12	0.6042	5	0.5868

TC28A12	0.2917	8	0.8090

TC28B01	0.2381	8	0.8345

TC28B07	0.4750	8	0.7213

TC28E09	0.8261	2	0.2873

TC29C07b	0.3913	5	0.6909

TC29H08	0.5909	3	0.5165

TC30D04	0.5217	6	0.6682

TC31C09	0.5417	3	0.5799

TC31G11a	0.4000	8	0.7663

TC31G11b	0.3750	4	0.7049

TC31H02	0.3636	6	0.7479

TC31H03	0.6190	4	0.5488

TC31H06	0.4545	7	0.7293

TC34E12	0.4737	4	0.6482

TC35F05	0.3182	7	0.7934

TC36C02	0.5789	5	0.6039

TC36C03	0.2778	6	0.7778

TC38A07	0.5000	4	0.5663

TC38F01	0.4375	7	0.7144

TC38H09	0.2667	7	0.8267

TC39A10	0.4474	9	0.7382

TC39B04	0.5000	4	0.6400

TC39C01	0.5238	6	0.6712

TC39E08	0.3750	9	0.7986

TC39F01	0.2353	8	0.8443

TC39F08	0.4762	7	0.7007

TC40D04	0.4667	5	0.7022

TC40E08	0.9565	2	0.0832

TC41A05	0.4706	6	0.6990

TC41A10	0.5238	8	0.6689

TC41A11b	0.1875	10	0.8750

TC41C05	0.9583	2	0.0799

TC41C11	0.4773	6	0.6911

TC42A02	0.4583	3	0.6424

TC42A05	0.5000	7	0.6710

**Mean**	**0.5056**	**5.51**	**0.6144**

List of the 78 polymorphic markers as screened against 22 accessions of *A. hypogaea*. Major allele frequencies, allele number amplified per locus, and gene diversity indices are also shown

The average number of alleles amplified per locus was 5.5, values of Gene Diversity (GD) were between 0.080 and 0.885, with an average of 0.614. Sixty-six markers were highly polymorphic with a GD of more than or equal to 0.5.

**Figure 1:**
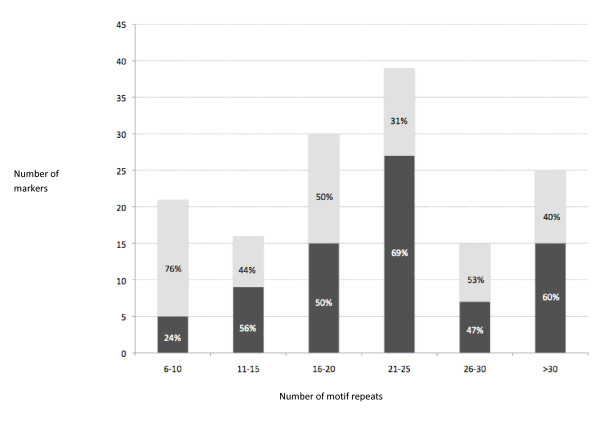
**Frequency of markers detected per repeat size class**. Frequency of markers developed in this study, polymorphic (dark grey), and monomorphic (light grey) per motif repeat number class. The percentage of polymorphic and monomorphic markers in each class is indicated on each bar of the graph.

Within cultivated peanut, markers with 21-25 motif repetitions were the most polymorphic (69%), followed by markers that amplified more than 30 motif repetitions (60%, most of the markers being composite or imperfect) (Figure 1). The lowest polymorphism was observed with short microsatellites, between 6-10 motif repetitions.

### Genic content

Thirty-six of the 135 marker sequences encoded putative proteins that had significant BLAST similarities to known predicted proteins of *Arabidopsis *and/or legumes (E-value < 1^e-07^, (Additional File 1: Table S1). Of the highly polymorphic markers (GD ≥ 0.5), 23% showed a significant BLAST similarity. This compares to 35% of the markers with GD < 0.5 that do not show significant BLAST similarity.

### Genetic relationships

**Figure 2:**
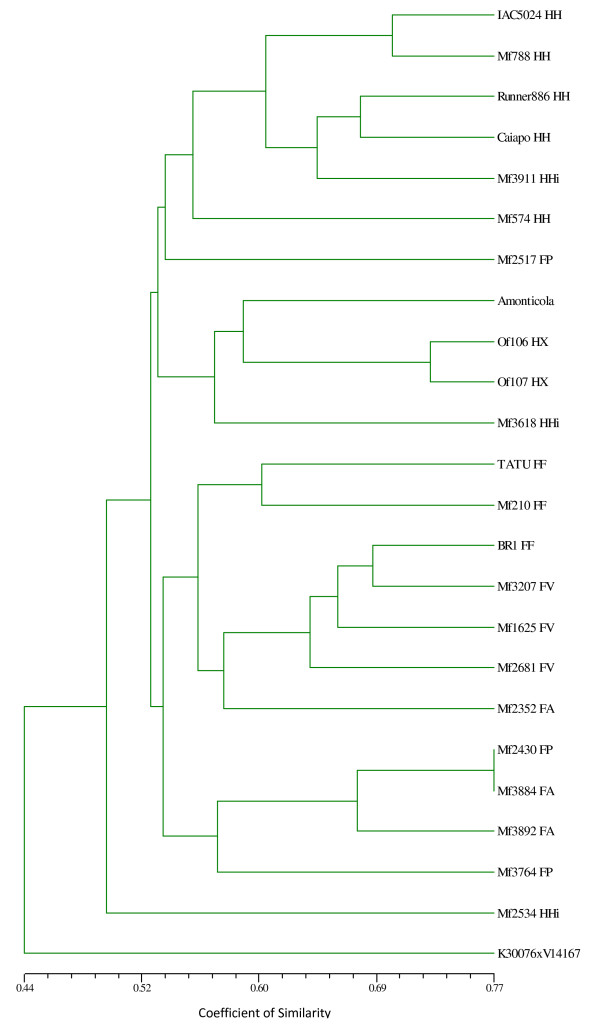
**Dendrogram based on the band-sharing distances of 24 *Arachis *genotypes, generated by UPGMA**. The letters, after each *A. hypogaea *accession number, refer to the subspecies, varieties, and type: FF-*fastigiata/fastigiata*; FV-*fastigiata/vulgaris*; FA-*fastigiata/aequatoriana*; FP-*fastigiata/peruviana*; HH-*hypogaea/hypogaea*; HHi-*hypogaea/hirsuta*; and HX-*hypogaea *Xingu type; an accession of *A. monticola *and a synthetic allotetraploid plant (K30076 × V14167)^4x^. Note, all accessions were distinguished, the highest point represented on the scale of similarity coefficient being 0.77.

Genetic similarities were estimated by the band-sharing coefficient [29] in pairwise comparisons of the 24 genotypes (Table 1), using 78 microsatellite loci. Genetic similarity values ranged from 0.42-0.77, considering the 22 *A. hypogaea *genotypes used. Therefore all the genotypes were differentiated. A dendrogram based on UPGMA was constructed for the 24 genotypes (Figure 2). Cluster analysis showed two main groups according to the subspecies. Within these groups, genotypes of the same botanical varieties tended to group together.

## Discussion

In spite of the considerable effort made by several research groups to develop molecular markers for cultivated peanut, the number of polymorphic markers available for this important crop is still limiting. One of the main challenges in the construction of linkage maps using populations derived from cultivated × cultivated crosses is the need to screen thousands of markers to obtain sufficient markers even for the construction of low density maps.

In this study we focused on the class of microsatellites that was shown to be the most highly polymorphic for cultivated peanut in a previous study, long TC repeats [10] For this, sequences were obtained from an enriched genomic library. For processing the sequences, the Staden software was used together with a module for the detection of microsatellites. Starting from a relatively large dataset of unassembled sequences, it was possible to quickly eliminate sequences that were similar to previously described markers, and assemble a compact database of microsatellite containing sequences. Using a naming convention of plasmid clones, it was possible to correctly assemble microsatellite-containing reads even when the only overlap between forward and reverse sequences were microsatellite repeats. This was particularly important for obtaining complete sequences when the repeats were long. For design of primer pairs, the program used took into account the quality values of consensus bases. This was reflected in the 100% success rate of amplification of the primer pairs.

Markers with 21-25 motif repetitions were the most polymorphic, while markers with shorter repeats tended to be less polymorphic. This general tendency agrees with previous studies and reinforces the view that long (21-25 motif repetitions) or composite TC microsatellites are probably the most polymorphic marker class for cultivated peanut. A slightly higher proportion of markers that were not polymorphic or less informative (GD < 0.5) showed significant similarities to protein encoding regions, probably reflecting a tendency for non-coding regions to be more polymorphic than coding regions. Overall 78 of the markers were polymorphic for the cultivated accessions and 66 of these had GD value of 0.5 or above.

Cluster analysis showed two main groups separating the two subspecies of *A. hypogaea*. Some tendency of grouping of genotypes according to their botanical varieties was also evident. The main exceptions were three accessions, Mf2517, Mf2352, and Mf2534, which clustered with no apparent reason. The upper group contained the five *hypogaea*/*hypogaea *genotypes and two of the three *hypogaea*/*hirsuta *genotypes. *Arachis monticola *and the two genotypes collected in the Xingu Indigenous Park also clustered in this group. The Xingu material has some morphological traits, especially in the pods, exceeding the previously variation described in cultivated peanut [30], but it seems to be closely related to *hypogaea*/*hypogaea *and *hypogaea*/*hirsuta *varieties. Our results also showed the great genetic similarity of the varieties *fastigiata *and *vulgaris*, which formed a subgroup, and *peruviana *and *aequatoriana*, which formed a separate subgroup. Some studies have shown that genotypes of the varieties *peruviana *and *aequatoriana *were more closely related to genotypes of the subspecies *hypogaea *than to the other two varieties (*fastigiata *and *vulgaris*) of subspecies *fastigiata *[8,17,31,32]. Our results, in contrast, corroborated the current taxonomical classification, despite the small number of genotypes included.

## Conclusion

In this study 146 new microsatellite markers were developed for *Arachis*. All of these markers are new and useful tools for genetics and genomics in *Arachis*, but in particular the set of 66 markers highly polymorphic for cultivated peanut are a significant step towards routine molecular breeding in this important crop.

## Methods

### Plant material and DNA extraction

For construction of an SSR-enriched genomic DNA library, the peanut genotype *A. hypogaea *subsp. *fastigiata *var. *fastigiata *cv. IAC-Tatu was used. For marker validation and genetic relationship analysis, the following panel was used: a set of 22 *A. hypogaea *genotypes representing all six botanical varieties, a synthetic allotetraploid (derived from a cross between *A. ipaënsis *and *A. duranensis*) and an accession of the tetraploid wild species, *A. monticola *(Table 1). Marker polymorphism was also assessed in parents of four mapping populations: *A. duranensis *K7988 × *A.stenosperma *V10309 [10,25], *A. ipaënsis *KG30076 × *A. magna *KG30097 [11], *A. hypogaea *subsp. *hypogaea *var. *hypogaea *cv. Runner IAC 886, and *A. hypogaea *subsp. fastigiata *var. vulgaris *cv. Fleur 11 × a synthetic amphidiploid [24] (Additional file 1).

Total genomic DNA was isolated from young leaves using the CTAB-based protocol described by Grattapaglia and Sederoff [33] modified by the inclusion of an additional precipitation step with 1.2 M NaCl. DNA quality and concentration were estimated on agarose gel electrophoresis and by spectrophotometry (Genesys 4 - Spectronic, Unitech, USA).

### Construction of SSR-enriched library

A genomic DNA library enriched for the dinucleotide repeats TC/AG was constructed as described by Moretzsohn [10]. About nine micrograms of DNA were digested with *Sau*3AI (Amersham Biosciences, UK) and electrophoresed in 0.8% low melting agarose gels to select fragments ranging from 200-600 bp. The selected fragments were purified from the agarose gels using phenol/chloroform, and ligated into *Sau*3AI specific adaptors (5'-cagcctagagccgaattcacc-3' and 5'-gatcggtgaaatcggctcaggctg-3'). The ligated fragments were hybridized to biotinylated (AG)_15 _oligonucleotides and isolated using streptavidin-coated magnetic beads (Dynabeads Streptavidin, Dynal Biotech, Norway). The eluted fragments were amplified using one adaptor-specific primer, cloned into the pGEM-T Easy vector (Promega, WI, USA) and transformed into XL1-Blue *E. coli *cells with blue/white selection (Invitrogen, CA, USA). Plasmid DNAs of the positive clones were isolated by the alkaline lysis method. Sequencing reactions were performed with T7 and SP6 primers and the Big-Dye Terminator Cycle Sequencing Kit, version 3.1 (Applied Biosystems, CA, USA) using the ABI Prism 377 automated DNA sequencer.

### SSR marker development and validation

Sequences were processed and assembled by using the Staden package [34] with the repeat sequence finding module TROLL [35] and Primer3 for primer design [36], using a module developed by Martins et al. [37]. Sequences with more than ten motif repeats were chosen for primer design. Some sequences with BLASTX hits to genes of interest were also included in spite of having fewer than ten motif repeats. The parameters for primer design were: (1) primer size ranging from 18 bp to 25 bp with an optimal length of 20 bp; (2) primer *Tm *(melting temperature) ranging from 57°C to 63°C with an optimal temperature of 60°C; and (3) GC content ranging from 40%-60%. Default values were used for the other parameters.

PCR reactions contained 10 ng of genomic DNA, 1 U of *Taq *DNA polymerase (Amersham Biosciences, UK), 1× PCR buffer (200 mM Tris pH 8.4, 500 mM KCl), 1.5-2.0 mM MgCl_2_, 200 μM of each dNTP, and 0.4 μM of each primer, in a final reaction volume of 10 μl. Amplifications were carried out in a PTC 100 thermocycler (MJ Research Inc., MA, USA). PCR conditions were: 96°C for 5 min, followed by 30 cycles of 94°C for 1 min, 48-62°C (annealing temperature depending on primer pair, see Additional file 1).

Total genomic DNA was isolated from young leaves using the CTAB-based protocol described by Grattapaglia and Sederoff [33] modified by the inclusion of an additional precipitation step with 1.2 M NaCl. DNA quality and concentration were estimated on agarose gel electrophoresis and by spectrophotometry (Genesys 4 - Spectronic, Unitech, USA).

### Construction of SSR-enriched library

A genomic DNA library enriched for the dinucleotide repeats TC/AG was constructed as described by Moretzsohn [10]. About nine micrograms of DNA were digested with *Sau*3AI (Amersham Biosciences, UK) and electrophoresed in 0.8% low melting agarose gels to select fragments ranging from 200-600 bp. The selected fragments were purified from the agarose gels using phenol/chloroform, and ligated into *Sau*3AI specific adaptors (5'-cagcctagagccgaattcacc-3' and 5'-gatcggtgaaatcggctcaggctg-3'). The ligated fragments were hybridized to biotinylated (AG)_15 _oligonucleotides and isolated using streptavidin-coated magnetic beads (Dynabeads Streptavidin, Dynal Biotech, Norway). The eluted fragments were amplified using one adaptor-specific primer, cloned into the pGEM-T Easy vector (Promega, WI, USA) and transformed into XL1-Blue *E. coli *cells with blue/white selection (Invitrogen, CA, USA). Plasmid DNAs of the positive clones were isolated by the alkaline lysis method. Sequencing reactions were performed with T7 and SP6 primers and the Big-Dye Terminator Cycle Sequencing Kit, version 3.1 (Applied Biosystems, CA, USA) using the ABI Prism 377 automated DNA sequencer.

### SSR marker development and validation

Sequences were processed and assembled by using the Staden package [34] with the repeat sequence finding module TROLL [35] and Primer3 for primer design [36], using a module developed by Martins et al. [37]. Sequences with more than ten motif repeats were chosen for primer design. Some sequences with BLASTX hits to genes of interest were also included in spite of having fewer than ten motif repeats. The parameters for primer design were: (1) primer size ranging from 18 bp to 25 bp with an optimal length of 20 bp; (2) primer *Tm *(melting temperature) ranging from 57°C to 63°C with an optimal temperature of 60°C; and (3) GC content ranging from 40%-60%. Default values were used for the other parameters.

PCR reactions contained 10 ng of genomic DNA, 1 U of *Taq *DNA polymerase (Amersham Biosciences, UK), 1× PCR buffer (200 mM Tris pH 8.4, 500 mM KCl), 1.5-2.0 mM MgCl_2_, 200 μM of each dNTP, and 0.4 μM of each primer, in a final reaction volume of 10 μl. Amplifications were carried out in a PTC 100 thermocycler (MJ Research Inc., MA, USA). PCR conditions were: 96°C for 5 min, followed by 30 cycles of 94°C for 1 min, 48-62°C (annealing temperature depending on primer pair, see Additional file 1).

Total genomic DNA was isolated from young leaves using the CTAB-based protocol described by Grattapaglia and Sederoff [33] modified by the inclusion of an additional precipitation step with 1.2 M NaCl. DNA quality and concentration were estimated on agarose gel electrophoresis and by spectrophotometry (Genesys 4 - Spectronic, Unitech, USA).

### Construction of SSR-enriched library

A genomic DNA library enriched for the dinucleotide repeats TC/AG was constructed as described by Moretzsohn [10]. About nine micrograms of DNA were digested with *Sau*3AI (Amersham Biosciences, UK) and electrophoresed in 0.8% low melting agarose gels to select fragments ranging from 200-600 bp. The selected fragments were purified from the agarose gels using phenol/chloroform, and ligated into *Sau*3AI specific adaptors (5'-cagcctagagccgaattcacc-3' and 5'-gatcggtgaaatcggctcaggctg-3'). The ligated fragments were hybridized to biotinylated (AG)_15 _oligonucleotides and isolated using streptavidin-coated magnetic beads (Dynabeads Streptavidin, Dynal Biotech, Norway). The eluted fragments were amplified using one adaptor-specific primer, cloned into the pGEM-T Easy vector (Promega, WI, USA) and transformed into XL1-Blue *E. coli *cells with blue/white selection (Invitrogen, CA, USA). Plasmid DNAs of the positive clones were isolated by the alkaline lysis method. Sequencing reactions were performed with T7 and SP6 primers and the Big-Dye Terminator Cycle Sequencing Kit, version 3.1 (Applied Biosystems, CA, USA) using the ABI Prism 377 automated DNA sequencer.

### SSR marker development and validation

Sequences were processed and assembled by using the Staden package [34] with the repeat sequence finding module TROLL [35] and Primer3 for primer design [36], using a module developed by Martins et al. [37]. Sequences with more than ten motif repeats were chosen for primer design. Some sequences with BLASTX hits to genes of interest were also included in spite of having fewer than ten motif repeats. The parameters for primer design were: (1) primer size ranging from 18 bp to 25 bp with an optimal length of 20 bp; (2) primer *Tm *(melting temperature) ranging from 57°C to 63°C with an optimal temperature of 60°C; and (3) GC content ranging from 40%-60%. Default values were used for the other parameters.

PCR reactions contained 10 ng of genomic DNA, 1 U of *Taq *DNA polymerase (Amersham Biosciences, UK), 1× PCR buffer (200 mM Tris pH 8.4, 500 mM KCl), 1.5-2.0 mM MgCl_2_, 200 μM of each dNTP, and 0.4 μM of each primer, in a final reaction volume of 10 μl. Amplifications were carried out in a PTC 100 thermocycler (MJ Research Inc., MA, USA). PCR conditions were: 96°C for 5 min, followed by 30 cycles of 94°C for 1 min, 48-62°C (annealing temperature depending on primer pair, see Additional file 1).

Total genomic DNA was isolated from young leaves using the CTAB-based protocol described by Grattapaglia and Sederoff [33] modified by the inclusion of an additional precipitation step with 1.2 M NaCl. DNA quality and concentration were estimated on agarose gel electrophoresis and by spectrophotometry (Genesys 4 - Spectronic, Unitech, USA).

### Construction of SSR-enriched library

A genomic DNA library enriched for the dinucleotide repeats TC/AG was constructed as described by Moretzsohn [10]. About nine micrograms of DNA were digested with *Sau*3AI (Amersham Biosciences, UK) and electrophoresed in 0.8% low melting agarose gels to select fragments ranging from 200-600 bp. The selected fragments were purified from the agarose gels using phenol/chloroform, and ligated into *Sau*3AI specific adaptors (5'-cagcctagagccgaattcacc-3' and 5'-gatcggtgaaatcggctcaggctg-3'). The ligated fragments were hybridized to biotinylated (AG)_15 _oligonucleotides and isolated using streptavidin-coated magnetic beads (Dynabeads Streptavidin, Dynal Biotech, Norway). The eluted fragments were amplified using one adaptor-specific primer, cloned into the pGEM-T Easy vector (Promega, WI, USA) and transformed into XL1-Blue *E. coli *cells with blue/white selection (Invitrogen, CA, USA). Plasmid DNAs of the positive clones were isolated by the alkaline lysis method. Sequencing reactions were performed with T7 and SP6 primers and the Big-Dye Terminator Cycle Sequencing Kit, version 3.1 (Applied Biosystems, CA, USA) using the ABI Prism 377 automated DNA sequencer.

### SSR marker development and validation

Sequences were processed and assembled by using the Staden package [34] with the repeat sequence finding module TROLL [35] and Primer3 for primer design [36], using a module developed by Martins et al. [37]. Sequences with more than ten motif repeats were chosen for primer design. Some sequences with BLASTX hits to genes of interest were also included in spite of having fewer than ten motif repeats. The parameters for primer design were: (1) primer size ranging from 18 bp to 25 bp with an optimal length of 20 bp; (2) primer *Tm *(melting temperature) ranging from 57°C to 63°C with an optimal temperature of 60°C; and (3) GC content ranging from 40%-60%. Default values were used for the other parameters.

PCR reactions contained 10 ng of genomic DNA, 1 U of *Taq *DNA polymerase (Amersham Biosciences, UK), 1× PCR buffer (200 mM Tris pH 8.4, 500 mM KCl), 1.5-2.0 mM MgCl_2_, 200 μM of each dNTP, and 0.4 μM of each primer, in a final reaction volume of 10 μl. Amplifications were carried out in a PTC 100 thermocycler (MJ Research Inc., MA, USA). PCR conditions were: 96°C for 5 min, followed by 30 cycles of 94°C for 1 min, 48-62°C (annealing temperature depending on primer pair, see Additional file 1) for 1 min, 72°C for 1 min, with a final extension for 10 min at 72°C. PCR products were separated by electrophoresis on denaturing polyacrylamide gels (6% acrylamide:bisacrylamide 29:1, 5 M urea in TBE pH 8.3), stained with silver nitrate [38].

### Data analyses

Number of alleles per locus, the range of fragment length and gene diversity (GD) were estimated for the polymorphic primers, using the program "Power Marker 3.25" [39]. Pairwise genetic similarities were estimated from the allelic data using the band-sharing coefficient of Lynch [29]. The resulting diagonal matrix was then submitted to cluster analysis using UPGMA ("unweighted pair-group method analysis"). In order to verify the consistency of the built dendrogram, the cophenetic correlation - r [40] was calculated. All these analyses were performed using the software NTSYS 2.21 [41].

## Competing interests

The authors declare that they have no competing interests.

## Authors' contributions

SEM participated in the experimental work, the identification of SSRs, primer design, and marker screening. EGG and DMTA participated in marker screening. DJB and MCM participated in the design and implementation of the study, supervision of the work and processing interpretation of the results. SCML-B participated in data analysis, microsatellite marker validation and drafted the manuscript. VCRA participated in the construction of SSR-enriched library. All authors read and approved the final manuscript.

## Supplementary Material

Additional file 1 **Table S1. Marker homologies and information**. The file provides information on all markers used in this work: marker name, primer sequence, SSR motif, GenBank number, PCR conditions, fragment length, sequence homologies and if the primers are polymorphic (P) or monomorphic (M) for parents of four mapping populations. Click here for file
